# Simultaneous Quantification of Opioids in Blood and Urine by Gas Chromatography-Mass Spectrometer with Modified Dispersive Solid-Phase Extraction Technique

**DOI:** 10.3390/molecules27196761

**Published:** 2022-10-10

**Authors:** Sara Yasien, Ejaz Ali, Mohsin Javed, Muhammad Muntazir Iqbal, Shahid Iqbal, Hamad Alrbyawi, Samar O. Aljazzar, Eslam B. Elkaeed, Ayed A. Dera, Rami Adel Pashameah, Eman Alzahrani, Abd-ElAziem Farouk

**Affiliations:** 1 University College of Pharmacy, University of the Punjab, Lahore 54590, Pakistan; 2 Department of Chemistry, School of Science, University of Management and Technology, Lahore 54770, Pakistan; 3 Department of Chemistry, School of Natural Sciences (SNS), National University of Science and Technology (NUST), H-12, Islamabad 46000, Pakistan; 4 Pharmaceutics and Pharmaceutical Technology Department, College of Pharmacy, Taibah University, Medina 42353, Saudi Arabia; 5 Department of Chemistry, College of Science, Princess Nourah bint Abdulrahman University, P.O. Box 84428, Riyadh 11671, Saudi Arabia; 6 Department of Pharmaceutical Sciences, College of Pharmacy, AlMaarefa University, Riyadh 13713, Saudi Arabia; 7 Department of Clinical Laboratory Sciences, College of Applied Medical Sciences, King Khalid University, Abha 61421, Saudi Arabia; 8 Department of Chemistry, Faculty of Applied Science, Umm Al-Qura University, Makkah 24230, Saudi Arabia; 9 Department of Chemistry, College of Science, Taif University, P.O. Box 11099, Taif 21944, Saudi Arabia; 10 Department of Biotechnology, College of Science, Taif University, P.O. Box 11099, Taif 21944, Saudi Arabia

**Keywords:** opioids, blood and urine, epsom salt, GC-MS, simultaneous

## Abstract

Common methodologies such as liquid-liquid extraction and solid-phase extraction are applied for the extraction of opioids from biological specimens i.e., blood and urine. Techniques including LC-MS/LC-MSMS, GC-MS, etc. are used for qualitative or quantitative determination of opioids. The goal of the present work is to design a green, economic, rugged, and simple extraction technique for famous opioids in human blood and urine and their simultaneous quantification by GC-MS equipped with an inert plus electron impact (EI) ionization source at SIM mode to produce reproducible and efficient results. Morphine, codeine, 6-acetylmorphine, nalbuphine, tramadol and dextromethorphan were selected as target opioids. Anhydrous Epsom salt was applied for dSPE of opioids from blood and urine into acetonitrile extraction solvent with the addition of sodium phosphate buffer (pH 6) and n-hexane was added to remove non-polar interfering species from samples. BSTFA was used as a derivatizing agent for GC-MS. Following method validation, the LOD/LLOQ and ULOQ were determined for morphine, codeine, nal-buphine, tramadol, and dextromethorphan at 10 ng/mL and 1500 ng/mL, respectively, while the LOD/LLOQ and ULOQ were determined for 6-acetylmorphine at 5 ng/mL and 150 ng/mL, respectively. This method was applied to real blood and urine samples of opioid abusers and the results were found to be reproducible with true quantification.

## 1. Introduction

Blood and urine are the most favorite specimens for the detection of drugs, especially opioids [1]. The quantity of drug intake can be determined more accurately in blood [2], while urine is considered good for qualitative identification of drugs and metabolites [3]. Both of these specimens possess complex matrix composition, but blood is a more complex matrix than urine due to the presence of fats and proteins [4]. Thus, sample preparation becomes an integral part of the analytical process while dealing with such complex biological matrices [5].

In 2003, QuEChERS extraction (pronounced as “catchers”) was first developed by Anastassiades, Lehotey, et.al. for the extraction of pesticides residue from food items [6]. Later, in 2004, it was coined as QuEChERS by Schenck and Hobbs [7]. Basically, it is solid phase dispersed liquid extraction in which a buffer of pH 6 plus acetonitrile is applied as extraction solvent and anhydrous MgSO_4_ is applied for phase separation between two miscible media [8]. Matrix constituents are salted out and water is trapped due to the addition of this anhydrous salt [9]. This method was developed for the extraction of pesticides from food items such as vegetable and fruits [10].

Any analyst will find it difficult to deal with biological matrices including blood, urine, gastric lavage, hair, nails, stomach contents, liver, etc. [11]. Thus, sample pretreatment becomes a necessary and crucial part in the analysis of such samples with complex matrix composition [12]. Matrix complexity in biological samples is due to the presence of proteins, carbohydrates, lipids, salts and various organic and inorganic compounds that may interfere with analyte of interest during analysis whether qualitative or quantitative [13]. Basically, signal suppression of an analyte of interest due to the presence of complex matrix interference is the problem that analytical scientist want to get rid of [14], while it is also not possible to analyze biological samples directly on any analytical tool, owing to the presence of matrix interference [15]. Hence, sample pretreatment involving some suitable extraction methodology becomes vital because without it, the whole analytical process proves to be useless [16]. In solid-phase extraction (SPE), cartridges packed with sorbents are used [17]. The first step of the extraction procedure involves cartridges preparation, after which the sample is loaded into them [18]. A specific buffer media compels the analytes to retain in sorbent [19]. After this, a specific organic elution solvent is applied in SPE cartridges that bring analytes out [20]. However, protein precipitation becomes necessary before sample loading into cartridges to avoid blockage in cartridges [21]. Liquid-liquid extraction is also an option for drugs extraction from biological specimens, such as blood, urine, liver and stomach contents [22]. In this method, some organic solvent is selected that is immiscible with aqueous phase of sample [23]. Specific buffered media has to be provided for transfer of analytes from aqueous media to organic media [24]. There is a drawback of liquid-liquid extraction because it consumes a large volume of organic solvents that are mostly harmful for environment [25]. Second, the extraction recovery of this method is lower and not suitable for trace amounts of analytes in biological specimens [26].

To determine multiple opioids in biological samples, especially blood and urine, many analytical tools has been applied [27]. High-performance liquid chromatography (HPLC) equipped with diode array detector (DAD) or fluorescence detector (FLD) has been used for qualitative or quantitative detection of some opioid [28]. However, the acceptability of such techniques eventually reduced due to some limitations, such as sensitivity, specificity and accuracy [29]. High-performance liquid chromatography coupled with mass spectrometer (HPLC-MS/HPLC-MSMS) replaced the previous mentioned technique [30]. Undoubtedly, this technology is very sensitive, specific and accurate, but it has some disadvantage, such as high cost, consuming a large number of organic solvents, frequent trouble shooting, non-availability of wide library database, etc. [31]. Gas chromatography-mass spectrometry (GC-MS) is also used for the qualitative and quantitative determination of opioids in blood and urine [32]. In scan mode, the GC-sensitivity MS’s is multiplicatively lowered while still allowing for the qualitative identification of any analyte. However, as it offers greater sensitivity and specificity for quantitative purposes, the ion monitoring (SIM) mode must be used [33]. Run times of GC-MS may vary according to set points of a method [34]. Various capillary columns, such as DB-1ms, DB-5ms, DB-35ms, etc., have been used in GC-MS with specific dimensions and stationary phase compositions [35]. Commonly used derivatization agents are BSTFA (*N*,*O*-Bis(trimethylsilyl)trifluoroacetamide), MTBSTFA (*N*-(tert-Butyldimethylsilyl)-*N*-methyltrifluoroacetamide) and MSTFA (N-Methyl-Ntrimethylsilyl-trifluoroacetamide) for analysis using GC-MS [36].

In this work, the modified dispersive solid phase extraction (dSPE) method has been developed in which acetonitrile extraction solvent is added in the sample (sample:acetonitrile = 1:4), along with addition of sodium phosphate buffer (pH 6). The addition of anhydrous MgSO_4_ dispersed the organic and aqueous media, removing maximum matrix residues. Next, the application of n-hexane pulled out non-polar interferences from the organic extract. The original dSPE method does not involve the addition of n-hexane for the removal of non-polar interference. Instead, EMR-lipid dSPE sorbent, which is costly as compared to n-hexane, is applied for removal of fatty compounds through affinity-based separation [37]. Gas chromatography-mass spectrometer (GC-MS) was chosen for simultaneous quantification of six opioids (tramadol, dextromethorphan, morphine, codeine, 6-acetylmorphine and nalbuphine) in selected ion monitoring (SIM) mode in blood and urine samples. After development and validation, the current method was applied on real blood and urine samples of opioid addicts. Some important information about opioid included in this work is mentioned in Table 1.

## 2. Methodology

### 2.1. Chemicals and Reagents

Certified reference materials (CRMs) included Morphine, Codeine, Nalbuphine, 6-acetyl-morphine, Dextromethorphan, Tramadol and Nalorphine (Cerilliant), Acetonitrile, (environmental grade 99.7%, Thermo Fisher Scientific, Waltham, MA, USA), Epsom salt anhydrous (99.5%, Alfa Aesar, Thermo Fisher Scientific), Sodium phosphate dibasic (≥99.0%, Sigma-Aldrich, Baden-Württemberg, Germany), Sodium chloride (≥99.0%, Thermo Fisher Scientific), n-Hexane (99.0%, Thermo Scientific, Waltham, MA, USA), Methanol (99.8%, Thermo Scientific), Distilled water (HPLC grade Thermo Scientific, Waltham, MA, USA) and BSTFA (*N*,*O*-Bis-trifluoroacetamide, Sigma-Aldrich, Baden-Württemberg, Germany).

### 2.2. Preparation of Standard Solutions

Stock standard solution of nalorphine internal standard was prepared in a screw-capped glass tube. For this, nalorphine CRM (1 mg/mL) was mixed in methanol to make a final volume of 10 mL with a concentration of 100 ug/mL and this was stored in the freezer at −10 °C. Stock standard solutions of each opioid (morphine, codeine, nalbuphine, 6-acetyl-morphine, dextromethorphan and tramadol) were prepared from CRM of the respective opioid (each having a concentration of 1 mg/mL) in methanol to make a final dilution volume of 10 mL with the concentration of 100 µg/mL. All these stock standards were stored in a freezer at −10 °C.

### 2.3. Preparation of Standards

Seven standards of known analyte concentration were prepared from a stock standard solution of each opioid in matrix-matched negative blood. Calibration range for 6-acetylmorphine was 5 ng/mL, 25 ng/mL, 50 ng/mL, 75 ng/mL, 100 ng/mL, 125 ng/mL and 150 ng/mL. While calibration range for all remaining opioids was 10 ng/mL, 100 ng/mL, 200 ng/mL, 500 ng/mL, 800 ng/mL, 1200 ng/mL and 1500 ng/mL.

### 2.4. Preparation of Quality Controls Checks

Matrix-matched blank blood was used to prepare positive and negative controls for the verification chromatogram of the final calibration curve for the quantification of target opioids in unknown samples. The concentration for 6-acetylmorphine made in positive control was 75 ng/mL and for all remaining targets, opioids were 500 ng/mL. Negative control was free from any target analyte (Figures S3 and S4).

### 2.5. Sample Collection and Storage

Blood and urine samples (Figures S1 and S2) of a drug addict were collected from rehabilitation center tubes containing potassium oxalate and sodium fluoride preservatives and stored in a refrigerator at 4–8 °C.

### 2.6. Extraction Procedure

Sample volume was kept at 1 mL for all including quality controls, standards of known concentrations and real samples before starting extraction. Consumable 15 mL screw-caped plastic tubes were used for the extraction process. First, 2 mL of sodium phosphate buffer of pH 6 was added, and then, 50 µL internal standard Nalorphine was spiked in all tubes. After that, 4 mL of extraction solvent acetonitrile and 100 mg NaCl were added to all tubes and agitated for 10 s on a vortex machine. Then, 2 g anhydrous Epsom salt was added to all tubes, agitated for 10 s on a vortex machine, rotated for 5 min on the auto rotator and centrifuged for 3 min at 3500 rpm. The supernatant of acetonitrile was transferred into a new tube with a pipette and the solvent was evaporated in a digital turbo vaporizer machine (the solvent is vaporized by two ways: hot water bath at bottom of tube and pressurized air strike at solvent surface on set point temperature plus pressure). Then, 50 µL acetonitrile was spiked in each tube and agitated for 10 s on a vortex machine. After that, 100 µL n-Hexane was spiked in each tube and agitated for 20 s for the removal of non-polar interfering species like cholesterol. Then, a drop-like layer of acetonitrile (at lower side due to being denser than n-hexane) was taken with the help of a micro pipette and transferred to a screw caped glass tube. Then, 25 µL of BSTFA (derivatizing agent) was spiked in each tube and heated for 20 min on a heat block at 60 °C. Finally, the sample was transferred into GC vials ready to run on GC-MS for quantitative analysis.

### 2.7. Instrument Programming

A gas chromatograph system (7890B GC, Agilent, Santa Clara, CA, USA) was hyphenated with an Inert Plus Mass spectrometer (5977B MSD, Agilent); the main programming included: front-inlet with split-less mode, inlet temperature 220 °C, Agilent J&W capillary column: length 15 m, internal diameter 0.25 mm, film thickness 0.25 µm, helium used as carrier gas at 8 psi pressure. Oven temperature ramping: initial temperature 100 °C with a hold of time 3 min, Ramp-1: 50 °C/min rise to 250 °C with a hold time of 2 min, Ramp-2: 60 °C/min rise to 310 °C with a hold time of 5 min, transfer line temperature 300 °C. Sample injection volume was 2 µL with a microliter syringe (capacity 5 µL).

In order to fragment the target analytes, a mass spectrometer (MS) was operated in SIM acquisition mode with gain factor 3 and an inert plus electron impact (EI) ionisation source under the following conditions: 250 °C for the ionisation source, 150 °C for the quadrupole, a fixed voltage of 70 eV, and a solvent delay time of 5 min.

### 2.8. Target Analytes with Respective SIM Ions

Three ions were selected for each target opioid and two ions for the internal standard. Respective ions for each analyte are given in Table 2.

### 2.9. Method Validation

Method validation guidelines of UNODC were followed including the parameters limit of detection (LOD), limit of quantification (LOQ), linearity range, accuracy, precision and interference study. For measurement of accuracy (% bias) and precision (% CV), triplicates of each concentration (lowest, medium and highest) were run for five days in different batches. Seven standards of known concentration were run to establish linearity. Only matrix interference was checked by spiking only internal standard (nalorphine) in analyte-free blood and urine samples.

Method ruggedness was checked through five parameters: rotator speed (rpm), rotation time (min), centrifuge speed (rpm), centrifuge time (min) and heat block temperature (°C) for derivatization. All these parameters were part of the sample preparation step. The objective of this activity was to determine any change in the final analytical results and effects of these five variables were observed over five days.

## 3. Results and Discussion

**Figure 1:**
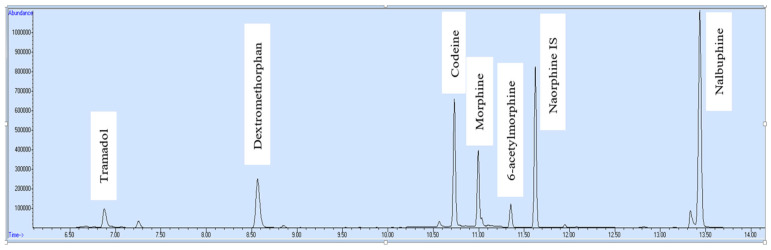
Standard—ion chromatogram.

Six opioids were determined in blood and urine samples in only 15 min run time on GC-MS. The labeled total ion chromatogram of opioid standard can be seen in Figure 1. The respective retention time of each analyte is shown in Table 3.

The developed method was validated against the parameters accuracy (% bias) and precision (% CV) for each target analyte, and their respective results are mentioned in Table 4. Three concentrations (lower, middle and high) were selected, and the triplicate of each concentration was run over five days in different batches.

During method validation, five parameters of method ruggedness were changed deliberately over five days, which are recorded in Table 5. Quantification results showed that such flexibility in the set points of these five parameters had no significant effect on the final analytical results.

The validation results of the newly developed method for the quantification of multiple opioids in blood and urine using GC-MS are mentioned in Table 6.

## 4. Application

**Figure 2:**
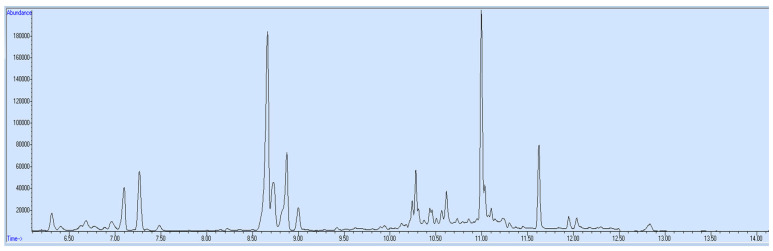
Blood sample—ion chromatogram.

**Figure 3:**
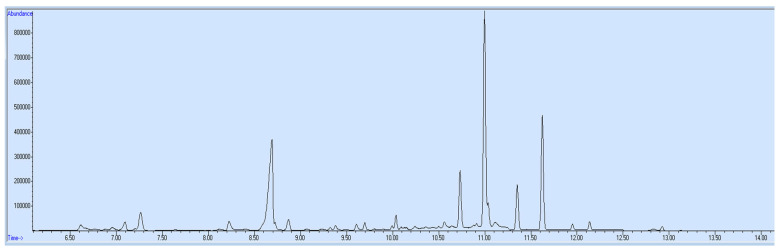
Urine sample—ion chromatogram.

The validated method was applied for quantification of target opioids in blood and urine specimens of an opioid addict. The addict was a chronic user of heroin and was brought to the rehabilitation center for treatment. His blood and urine specimens were collected for drug examination. After extraction by this novel technique, the samples were run on GC-MS. Results (Figure 2 and Figure 3) revealed that the blood sample contained 125 ng/mL morphine and 203 ng/mL dextromethorphan, while the urine sample contained 158 ng/mL morphine, 128 ng/mL 6-acetylmorphine and 264 ng/mL dextromethorphan. After a medical interview by the consultant, the drug addict admitted that he had taken heroin and cough syrup.

## 5. Conclusions

The current method of extraction is green, economical, simple and a time saver. Simultaneous quantification of morphine, codeine, 6-acetylmorphine, nalbuphine, tramadol and dextromethorphan on GC-MS was made possible with a short run time of only 14 min after method validation. For all analytes, recovery was good, accuracy (% bias) was less than 1 and precision (% CV) was less than 5. Linearity range for 6-acetylmorphine was 5–150 ng/mL, while for all other target opioids it was 10–1500 ng/mL, with an r^2^ value greater than 0.985. While they were 10 ng/mL for all other target opioids, the LOD and LOQ for 6-acetylmorphine were 5 ng/mL. This analytical method has the potential to accurately quantify the mentioned opioids in blood and urine samples of opioid addicts as well as opioid victims in clinical and forensic laboratories.

## Figures and Tables

**Table 1:** Target opioids with their half-life, analgesic duration and protein binding.

Opioid	Half Life (h)	Analgesic Duration (h)	Protein Binding (%)
Morphine	1.3–6.7	4–5	35
Codeine	1.2–3.9	3–4	7–25
Tramadol	4.3–6.7	2.6–2.9	15–20
Nalbuphine	1.9–7.7	3–6	25–40
Dextromethorphan	3–4	2–3	60–70

**Table 2:** Target opioids with their respective SIM ion.

Opioid Name	Quantifier Ion (m/z)	Qualifier Ion 1 (m/z)	Qualifier Ion 2 (m/z)
Tramadol	335	320	245
Dextromethorphan	271	270	214
Codeine	371	178	234
Morphine	429	236	287
6-Acetylmorphine	399	287	340
Nalbuphine	574	518	428
Nalorphine (IS)	455	414	-

**Table 3:** Target opioid with relevant retention time on GC-MS.

Opioid Name	Retention Time (minutes)
Tramadol	6.88
Dextromethorphan	8.56
Codeine	10.73
Morphine	10.99
6-Acetylmorphine	11.35
Nalbuphine	13.43
Nalorphine (IS)	11.62

**Table 4:** Accuracy (% bias) and precision (% CV) of all target opioids.

Target Opioid	Actual Concentrations (µg/L) of Three Standards	Accuracy (% bias) Range	Precision (% CV) Range
**Tramadol**	10	0.02–0.253	0.418–2.40
	800	0.081–0.133	0.10–0.016
	1500	0.142–0.237	0.009–0.015
**Dextromethorphan**	10	0.041–0.133	0.41–1.24
	800	0.081–0.133	0.01–0.015
	1500	0.203–0.268	0.013–0.017
**Codeine**	10	0.028–0.342	0.287–3.26
	800	0.081–0.133	0.01–0.016
	1500	0.142–0.257	0.008–0.015
**Morphine**	10	0.020–0.214	0.287–326
	800	0.081–0.133	0.01–0.016
	1500	0.142–0.257	0.008–0.015
**6-acetylmorphine**	5	0.041–0.253	0.379–2.65
	100	0.081–0.133	0.01–0.016
	150	0.142–0.237	0.009–0.015
**Nalbuphine**	10	0.077–0.315	0.006–5.67
	800	0.59–0.247	0.057–0.253
	1500	0.124–0.132	0.080–0.20

**Table 5:** Parameters observed for method ruggedness.

Days	Rotator Speed (rpm)	Rotation Time (min)	Centrifuge Speed (rpm)	Centrifugation Time (min)	Derivatisation Temperature (°C)
**Day1**	15	20	2000	15	65
**Day2**	20	16	2500	12	70
**Day3**	25	12	3000	9	75
**Day4**	30	8	3500	6	80
**Day5**	35	4	4000	3	85

**Table 6:** Summarized results of method validation parameters.

Parameter	Actual Results
**No. of target opioid**	6
**Extraction time per sample**	Almost 35 min
**GC-MS** **run time**	14 min
**Limit of detection (lod)**	5ng/mL for 6-acetylmorphine
**Limit of quantitation (loq)**	5–150 ng/mL for 6-acetylmorphine, and 10–1500 ng/mL for all other opioids
**Linearity**	5–150 ng/mL, r^2^ ≥ 0.985 for 6-acetylmorphine, and 10–1500 ng/mL for all other opioids, r^2^ ≥ 0.985
**Precision (% cv)**	It was less than 5 for all analytes
**Accuracy (% bias)**	It was less than 1 for all analytes
**Recovery**	Greater than 80%
**Ruggedness**	The method proved to be rugged
**Interference**	Absence of any interference in quantification

## Data Availability

The data presented in this study are available in Supplementary Materials.

## Supplementary Materials

The following are available online at https://www.mdpi.com/article/10.3390/molecules27196761/s1, Figure S1: Dispersed phase in blood sample; Figure S2: Dispersed phase in the urine sample; Figure S3: Positive QC-Total ion chromatogram; Figure S4: Negative QC-Total ion chromatogram.
