# Supplementary material for: Simultaneous Quantification of Opioids in Blood and Urine by Gas Chromatography-Mass Spectrometer with Modified Dispersive Solid-Phase Extraction Technique
Source: Molecules. 2022 Oct 10;27(19):6761. doi: 10.3390/molecules27196761 (PMC9570840; doi:10.3390/molecules27196761)
Supplement: Supplementary file 1 [file molecules-27-06761-s001.zip › molecules-1936779-supplementary.pdf]

Supporting Information

Simultaneous quantification of opioids in blood and urine by Gas chromatography-Mass spectrometer with modified dispersive solid-phase extraction technique

Sara Yasien[1], Ejaz Ali[1*], Mohsin Javed[2], Muhammad Muntazir Iqbal[2], Shahid Iqbal[3]*, Hamad Alrbyawi[4], Samar O. Aljazzar[5], Eslam B. Elkaeed[6], Ayed A. Dera[7], Rami Adel Pashameah[8], Eman Alzahrani[9], Abd-ElAziem Farouk[10]

[1]*University College of Pharmacy, University of the Punjab, Lahore, Pakistan.*
[2]*Department of Chemistry, School of Science, University of Management and Technology, Lahore 54770, Pakistan.*
[3]*Department of Chemistry, School of Natural Sciences (SNS), National University of Science and Technology (NUST), H-12, Islamabad, 46000, Pakistan.*
[4]*Pharmaceutics and Pharmaceutical Technology Department, College of Pharmacy, Taibah University, Medina 42353, Saudi Arabia*
[5]*Department of Chemistry, College of Science, Princess Nourah bint Abdulrahman University, P.O. Box 84428, Riyadh 11671, Saudi Arabia.*
[6]*Department of Pharmaceutical Sciences, College of Pharmacy, AlMaarefa University, Riyadh 13713, Saudi Arabia.*
[7]*Department of Clinical Laboratory Sciences, College of Applied Medical Sciences, King Khalid University, Abha, Saudi Arabia.*
[8]*Department of Chemistry, Faculty of Applied Science, Umm Al-Qura University, Makkah 24230, Saudi Arabia.*
[9]*Department of Chemistry, College of Science, Taif University, P.O. Box 11099, Taif 21944, Saudi Arabia.*
[10]*Department of Biotechnology College of Science, Taif University, P.O. Box 11099, Taif 21944, Saudi Arabia.*

To whom corresponding should be addressed

shahidgcs10@yahoo.com (Shahid Iqbal) and ejaz.pharmacy@pu.edu.pk (Ejaz Ali)

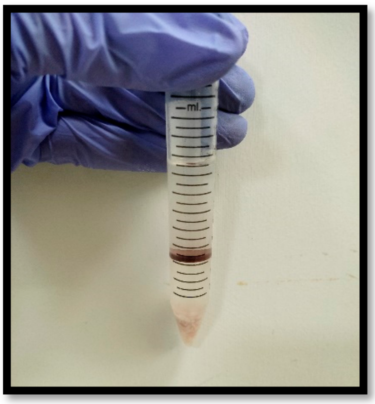

Figure. S1 *Dispersed phase in blood sample*

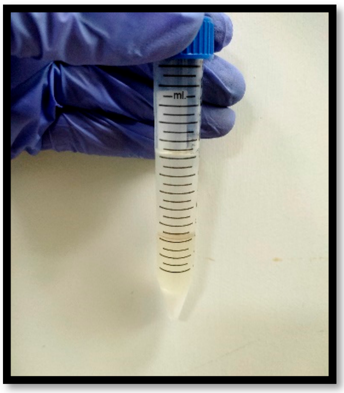

Figure. S2 *Dispersed phase in the urine sample*

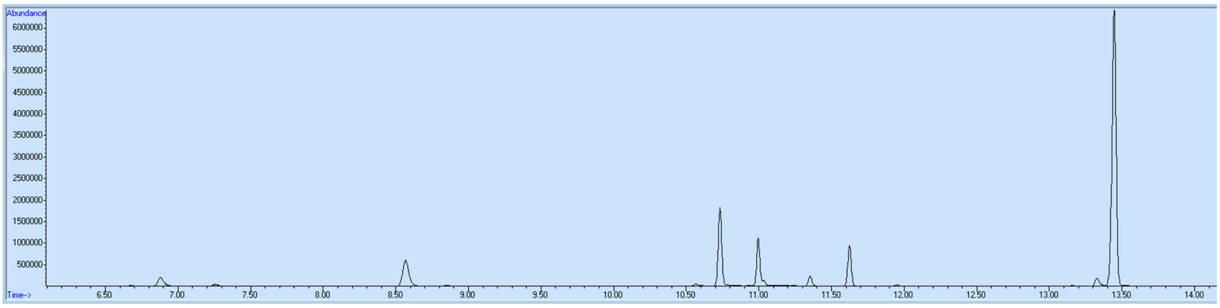

Figure. S3 *Positive QC-Total ion chromatogram*

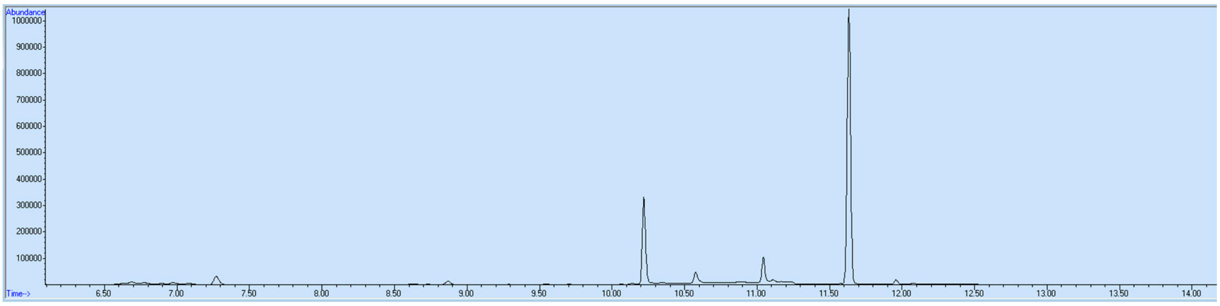

Figure. S4 *Negative QC-Total ion chromatogram*
